# Exploring the determinants of distress health financing in Cambodia

**DOI:** 10.1093/heapol/czz006

**Published:** 2019-10-23

**Authors:** Por Ir, Bart Jacobs, Augustine D Asante, Marco Liverani, Stephen Jan, Srean Chhim, Virginia Wiseman

**Affiliations:** 1 National Institute of Public Health, Lot No. 80, Street 289, Phnom Penh, Cambodia; 2 Social Health Protection Programme, Deutsche Gesellschaft für Internationale Zusammenarbeit (GiZ), Lot No. 80, Street 289, Phnom Penh, Cambodia; 3 School of Public Health & Community Medicine, University of New South Wales, Sydney, New South Wales, Australia; 4 Department of Global Health and Development, London School of Hygiene & Tropical Medicine, 15-17 Tavistock Pl, Kings Cross, London, UK; 5 The George Institute for Global Health, University of New South Wales, 1 King St Newtown, New South Wales, Australia; 6 Kirby Institute, University of New South Wales, Wallace Wurth Building, High St, Kensington NSW, Australia

**Keywords:** Healthcare, borrowing, indebtedness, distress financing, determinant, Cambodia

## Abstract

Borrowing is a common coping strategy for households to meet healthcare costs in countries where social health protection is limited or non-existent. Borrowing with interest, hereinafter termed distress health financing or distress financing, can push households into heavy indebtedness and exacerbate the financial consequences of healthcare costs. We investigated distress health financing practices and associated factors among Cambodian households, using primary data from a nationally representative household survey of 5000 households. Multivariate logistic regression was used to determine factors associated with distress health financing. Results showed that 28.1% of households consuming healthcare borrowed to pay for that healthcare with 55% of these subjected to distress financing. The median loan was US$125 (US$200 for loans with interest and US$75 for loans without interest). Approximately 50.6% of healthcare-related loans were to pay for the costs of outpatient care in the past month, 45.8% for inpatient care and 3.6% for preventive care in the past 12 months. While the average period to pay off the loan was 8 months, 78% of households were still indebted from loans taken over 12 months before the survey. Distress financing is strongly associated with household poverty—the poorer the household the more likely it is to borrow, fall into debt and unable to pay off the debt—even for members of the health equity funds, a national scheme designed to improve financial access to health services for the poor. Other determinants of distress financing were household size, use of inpatient care and outpatient consultations with private providers or with both private and public providers. In order to ensure effective financial risk protection, Cambodia should establish a more comprehensive and effective social health protection scheme that provides maximum population coverage and prioritizes services for populations at risk of distress financing, especially poorer and larger households.

**Key Messages**

- In Cambodia, where social health protection is limited a large proportion of households experience ‘distress financing’—borrowing money with interest to pay for healthcare—potentially rendering them further indebted and exacerbating the financial consequences of healthcare costs.
- Household poverty is a key determinant of distress financing, even for households covered by the health equity fund, a national scheme designed to provide financial risk protection to poor households.
- In order to minimize distress financing, efforts should focus on establishing a comprehensive social health protection system that provides effective financial risk protection and maximizes population coverage while prioritizing selected services and population groups such as the poor and larger households.

## Introduction

In the absence of social health protection mechanisms, paying out-of-pocket (OOP) for healthcare can have serious adverse consequences for patients and their families. To pay for health services, people with little cash on hand have to resort to coping mechanisms such as using savings, borrowing money, selling assets, reducing food consumption, withdrawing children from school or foregoing further medical care (McIntyre *et al.*, 2006). Borrowing (with or without interest and/or selling of assets) to finance OOP expenses for healthcare has been termed distress or hardship financing (Joe, 2015). Distress financing mainly affects poor people as found in Argentina, India, Tanzania and rural China (Huffman *et al.*, 2011) and can represent a long-term burden for families with limited financial resources. While the sale of assets, especially productive ones such as livestock or agricultural land, can impose or aggravate poverty, this coping mechanism is less common than borrowing (Hoque *et al.*, 2015; Joe, 2015; Quintussi *et al.*, 2015).

Borrowing is a common coping strategy used by households to meet their healthcare costs in developing countries (Flores *et al.*, 2008). Approximately 22% of households in 40 low- and middle-income countries (LMICs) resort to borrowing to pay for healthcare services (Kruk *et al.*, 2009). In South Asia, many families are forced to work for little or no payment to repay loans (Daru *et al.*, 2005). In Vietnam, indebtedness is a common reason for women to become commercial sex workers or withdraw their children from school (Busza, 2004; Nguyen *et al.*, 2012). Borrowing can take different forms with none or various levels of interest, depending on the nature of the loan, socio-economic status (SES) of the borrower and time period for repayment (Ir *et al.*, 2012). Often interest rates can be considerable, as shown in Bangladesh where money lenders charged on average 8% per month or 96% per year (Hoque *et al.*, 2015). Such high-interest rates force families to take additional loans in order to pay for the earlier ones (Nguyen *et al.*, 2012). In contrast, Chichaibelu and Waibel (2017) found that multiple borrowing led to over-indebtedness in Thailand, but did not find evidence that loans were taken to pay off other debts. Thus, loans with high-interest rate impose greater economic hardship than those without interest by increasing the total amount of money that must be repaid, often making it impossible to service the principal loan (Quintussi *et al.*, 2015).

The ability to access informal loans, to borrow with low or no interest rate or without collateral is influenced by the prevailing degree of social capital which has been defined as ‘the information, trust, and norms of reciprocity inherent in one's social networks’ (Woolcock, 1998). In Cambodia, it has been shown that only 2% of patients who were able to borrow from relatives to pay for healthcare costs incurred interest compared with 32% who borrowed from neighbours and 100% who borrowed from private lenders or formal institutions (Jacobs *et al.*, 2007a). In Indonesia, social networks were found to be an important source of information for credit opportunities (Okten and Osili, 2004) while in southeast India poor households’ ability to access credit with low interest rates was influenced by their social relationships (Bhukuth *et al.*, 2018). The requirement for collateral, and thus ability to access credit by poor households, was inversely correlated with the intensity of social bonds between lenders and borrowers (Feder *et al.*, 1988; Lainez, 2014). The ability to borrow from relatives in Cambodia has drastically decreased over time. Whereas in 2009, 20.8% obtained loans from relatives, only 10.9% did so in 2014. Instead, borrowing from formal lending institutions like banks and microfinance institutions increased from 47.3% to 69.9% during the same period (National Institute of Statistics, 2015).

Apart from the socio-economic consequences, indebtedness may have a negative impact on physical and mental health, further worsening the financial situation due to increased demands for healthcare coupled with inability to work. In the USA, it was found that indebtedness and difficulties with loan repayment were associated with higher perceived stress, depression and worse self-reported general health (Skinner e*t al.*, 2004; Sweet *et al.*, 2013; Turunen and Hiilamo, 2014; Clayton e*t al.*, 2015). Stress causes physiological changes that are instrumental in several disease processes, especially diseases of the metabolic and cardiovascular systems, but can also influence health indirectly by modifying health-related behaviours such as diet, physical activity and substance abuse (McEwen, 1998, 2008). Eisenberg-Guyot *et al.* (2018) found that people with short-term high-interest loans—the so-called ‘fringe banking’—had a 38% higher likelihood of having poor health. In their review of the health effects of indebtedness, Turunen and Hiilamo (2014) found a multiplier effect of high-interest debt on health, especially amongst those least likely to repay. Illness and indebtedness can be mutually reinforcing in a vicious cycle, where more illness necessitates more treatments that in turn require more money and lead to further borrowing and stress. The stress of being in debt in turn induces illness (Turunen and Hiilamo, 2014).

Borrowing to pay for healthcare is common in Cambodia. The 2014 socio-economic survey found that 7.5% of households had outstanding debt or liabilities as a result of borrowing to pay for healthcare (National Institute of Statistics, 2013). Additionally, the practice of multiple loans has also been documented although the extent of this practice in the country is not well known (Liv, 2013). The 2014 Cambodian Demographic and Health Survey found that 12.4% of people with an illness borrowed money to pay for treatment and the proportion of people borrowing varies according to the amount of healthcare costs: only 2.4% of people whose healthcare costs were US$1 or less resorted to borrowing while 27.9% of people with a bill of US$100 or more borrowed funds (National Institute of Statistics, 2015). What is not reflected in these statistics is the frequency of health spending. Usually people with chronic conditions that require regular use of health services may incur only a small cost per episode, however, over time this could accumulate to a substantial cost and force them to borrow in order to meet such recurring expenditure (Goryakin and Suhrcke, 2014).

A number of studies have begun to explore in detail borrowing trends in Cambodia, including different typologies of borrowing and repayment methods, and their implications for health and poverty (Van Damme *et al.*, 2004). For example, it is known that interest-free loans tend to be provided by relatives or friends and, therefore, are easier to repay and impose less hardship on the household (Jacobs *et al.*, 2007b). However, when this option is not available, many Cambodians, especially poor households with limited access to formal creditors, take out loans with high-interest rates from informal money lenders, with significant implcations for their livelihoods (Ir *et al.*, 2012). A better understanding of these practices and trends will help in designing interventions to prevent distress financing and mitigate its effects on poor households. Our aim in this article is to explore the borrowing practices and the determinants of distress financing among 5000 randomly selected households in Cambodia. Following Binnendijk *et al.* (2012), we consider only distress financing for loans with interest since these are more likely to be associated with vulnerable population groups, such as those who cannot rely on social networks, and thus, are more prone to distress financing.

### Conceptual framework

Relatively few studies have investigated factors associated with borrowing to pay for health services. Kruk *et al.* (2009) showed that borrowing was more frequent among larger households, households of lower SES and households that incurred higher health expenditure. Similarly, Leive and Xu (2008) found that high-income households were least likely to borrow compared with lower income households and households with higher inpatient expenses were significantly more likely to borrow and deplete assets compared with those financing ambulatory care or routine medical expenses. A study in India (Joe, 2015) found that distress financing was mainly associated with hospitalization, healthcare for the elderly, treatment seeking for non-communicable diseases, and use of private sector providers. Another study in the same country found that the aggregated costs of ambulatory care were substantial (Berman *et al.*, 2010), an issue further documented by Binnendijk *et al.* (2012) who also found that maternity care leads to distress financing.

Other studies, while not directly assessing determinants of borrowing, have measured ‘catastrophic healthcare payments’. Catastrophic payments concern OOP expenses that exceed a certain threshold of a household’s ability-to-pay (Wagstaff *et al.*, 2017). Catastrophic payments can be considered a proxy for borrowing or distress financing. For example, Kastor and Mohanty (2018) found that 75% of people in India who resorted to distress financing also had catastrophic health expenses. In India, Prinja *et al.* (2016) found no difference in risks for catastrophic expenditures and distress financing while Madan *et al.* (2015) found distress financing to be a reliable indicator for catastrophic health expenses amongst tuberculosis patients.

**Figure 1:**
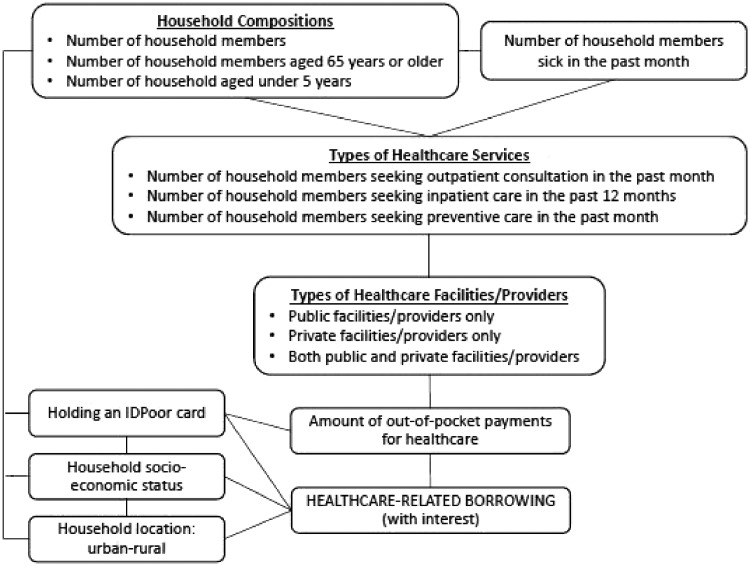
Potential factors associated with healthcare-related borrowing.

In summary, the potential factors associated with healthcare-related borrowing (in particular borrowing with interest) include household SES, household location (urban-rural), number of household members (household size), number of elderly members aged 65 years or older, number of children under-five years of age, number of members sick in the past month, type of healthcare service received/used (outpatient consultation, inpatient care, preventive care such as vaccination, family planning, antenatal care, delivery and postnatal care) and the location of services utilized. These factors can be directly associated with healthcare-related borrowing or indirectly through their interactions as illustrated in Figure 1. Characteristics that may reduce the risk of distress financing are place of residence whereby urban people can access cheaper healthcare (Fernandes Antunes *et al.*, 2018), SES whereby richer people have more cash at hand and IDPoor card holders who can access free healthcare at the point of access at public health facilities under the health equity funds (HEF). The latter is a nationwide social health protection scheme that reimburses health providers the user fees for services provided to eligible poor patients (Flores *et al.*, 2013).

## Methods

### Data

A nationally representative cross-sectional survey of 5000 households was conducted in early 2016. The households were randomly selected through a two-stage stratified cluster sampling method. Based on a national sampling frame, we first randomly selected 200 clusters (village or enumeration areas) using probability proportional-to-size method. In each cluster, 25 households were randomly selected from the list of households using simple random sampling. The sample was stratified be urban and rural areas in line with the latest inter-census results from 2013 that indicated an urban population of 21.4% of total population, with the capital accounting for 11.8% of the total population (National Institute of Statistics, 2013). More details on the sampling method are provided in the previously published research protocol (Wiseman *et al.*, 2017).

### Outcome variable

The outcome variable of interest in the present study is the prevalence of households reporting at least one incidence of healthcare utilization and borrowing money with interest to pay for that healthcare.

### Explanatory variables

The explanatory variables in this study include household demographics such as the location of the household, household size, number of children under 5 years old, and number of elderly aged 65 years or older in the household and number of household members reporting being sick in the month preceding the survey. We performed an asset-based principal component analysis to construct an index of household SES. Households were then ranked into wealth quintiles based on the asset index. The lowest quintile (first quintile) represented the poorest 20% of households and the highest quintile (fifth quintile) represented the richest 20%. Health service utilization was measured using three key variables: the number of household members seeking outpatient consultation in the past month, number of members seeking inpatient care (hospitalization) in the past 12 months, and number of members seeking preventive care in the past 12 months. We compared borrowing by type of healthcare provider—at public facilities only, private facilities only and both public and private facilities. Public health facilities included national hospitals, referral hospitals and health centres while private facilities included private hospitals, clinics, pharmacies, cabinets, private clinics in the homes of doctors and nurses or home visits at private doctors and nurses. Finally, the lump sum OOP payment for healthcare made by all household members in the past year was based on self-reported OOP payment by household heads. The costs included service fees, medicines, laboratory tests, informal payments (gift to provider, bribe, etc.), room fee for patients, food, accommodation, travel and all other expenses incurred by relatives’ accompanying patients.

The proportion of households borrowing money to pay for healthcare was calculated by dividing the total number of households that borrowed money to pay for healthcare by the total number of households that utilized healthcare. Descriptive statistics including proportions, means with standard deviation (for continuous variables close to normal distribution) and median with interquartile rank (for continuous variables that were skewed) were used to describe the characteristics of the households. Variables which were potentially associated with borrowing were identified using bivariate analysis. All potential predicting variables with an overall *P*-value <0.25 were included in the multivariate logistic regression. Stata software (Stata Corp, USA, version 14.1) was used to analyse the data.

## Results

Of the total 5000 surveyed households, 4996 reported at least one episode of healthcare utilization by their members: outpatient consultation (OPD) in the past month; inpatient admission (IPD) and use of preventive services (vaccination, family planning, antenatal care, delivery and postnatal care) in the 12 months preceding the survey. The total number of household members was 24 739.

### Household characteristics and healthcare seeking

Table 1 describes key characteristics of the 4996 households with at least one episode of healthcare utilization. Of these households, 80.1% were located in rural areas and the remaining 19.9% in urban areas. The median number of household members was 5 with 42.3% of the households having a child aged less than 5 years and 26.5% having an elderly person aged 65 years or older. Of the 4996 households, 85.8% had at least one member that reported being ill or injured in the month preceding the survey, 45.8% reported to have at least one member seeking outpatient consultation in the past month, and 22.6% and 38.8% seeking inpatient and preventive care in the past 12 months, respectively. In terms of type of provider, nearly 64% of households had at least one member who had used a private facility for OPD, 7.5% of households had at least one member who had sought care in a public facility, and 14.5% had at least one member using both public and private facilities. The proportion of households with a member hospitalized at a public health facility was 10.5%, while 12% had a member hospitalized at a private facility. For preventive care, the public sector accounted for 25.9% of the households compared with only 12.6% of households with members who sought such care in the private sector.

**Table 1:** Descriptive statistics of sampled households in Cambodia (2016)

	Total households (*N* = 4996)	
	Frequency	Percent
Household location		
Urban	993	19.9
Rural	4005	80.1
SES (classified into five quintiles)		
1st (poorest)	1033	20.7
2nd	972	19.5
3rd	1081	21.6
4th	911	18.2
5th (richest)	999	20.0
Household size		
Mean	5	
Less than 5	2281	45.9
5 or more	2687	54.1
Number of household members aged under 5 years		
0	2882	57.7
1	1225	24.5
2 or more	889	17.8
Number of household members aged 65 years or older		
0	3674	73.5
1	943	18.9
2 or more	379	7.6
Number of household members who were sick or injured in the past month		
0	707	14.2
1	1816	36.4
2	1332	26.7
3 or more	1140	22.8
Number of household members with at least one outpatient consultation in the past month		
0	2706	54.2
1	726	14.5
2	771	15.4
3 or more	793	15.9
Number of household members with at least one inpatient care in the past 12 months		
0	3869	77.4
1	964	19.3
2	119	2.4
3 or more	44	0.9
Number of household members with at least one preventive care utilization in the past 12 months		
0	3057	61.2
1	1284	25.7
2	550	11.0
3 or more	105	2.1
Type of health facility for outpatient consultation by household member(s) in the past month		
Did not use	720	14.4
Public	374	7.5
Private	3178	63.6
Both	724	14.5
Type of health facility for last inpatient care by household member(s) in the past 12 months		
Did not use	3872	77.5
Public	523	10.5
Private	601	12.0
Type of health facility for last preventive care by household member(s) in the past 12 months		
Did not use	3072	61.5
Public	1295	25.9
Private	629	12.6
OOP payment (in US$) for healthcare in the past 12 months		
Median	203	
<100	1792	35.9
101–200	690	13.8
201–400	1056	21.2
≥400	1458	29.2
Households with an IDPoor (HEF) card		
Yes	1172	23.5
No	3819	76.4
Missing	5	0.1
Households borrowing for healthcare		
Borrowing with interest	771	15.4
Borrowing without interest	632	12.7
Did not borrow	3593	71.9

The median OOP payment for healthcare in the past 12 months per household was US$203, ranging from US$48 to US$600, with 35.9% of households spending less than US$100 and 29.2% spent US$400 or more. The proportion of households reported to hold an IDPoor card that entitled them to financial support from a HEF was 23.5%. Of the households with at least one episode of healthcare utilization, 28.1% reported having to borrow money to pay for healthcare, including 15.4 with interest and 12.7% without.

### Borrowing characteristics

Table 2 describes the key characteristics of healthcare-related borrowing (loans), including distress financing (loans with interest, third column of the table) and those without (fourth column of the table). Of the healthcare-related loans, 80% were taken purposively for paying for healthcare while the other 20% was taken for multiple purposes including to pay for healthcare. The proportion of loans used to pay for outpatient care and inpatient care was 50.6% and 45.8%, respectively, compared with only 3.6% for preventive care, and there is no significant difference between borrowing with interest and without interest. The median loans per household were US$125, with an interquartile range of US$50–US$300. Loans were larger where interest was charged (US$200) compared with loans without interest (US$75). For 37.6% of the households, the borrowed amount was less than US$100, and US$400 or over for 22.8% of the households. Among households borrowing with interest, the average period to pay off the loan was 8 months (ranging from 3–12 months) with 41.7% and 46.6% of loans due in under 6 and 12 months, respectively. At the time of the survey, 21.3% of households that took out a loan in the previous 12 months had already paid it off while the remaining 78% of the households were still indebted. This proportion of indebtedness is similar between the households borrowing with interest and those borrowing without interest.

**Table 2:** Healthcare*-*related borrowing practices of sampled households in Cambodia (2016)

	Total households (*N* = 4996)		
	Households borrowing for healthcare	Households borrowing with interest (distress financing)	Households borrowing without interest
	% (*n*)	% (*n*)	% (*n*)
Mode of borrowing for healthcare			
Specifically to pay for health care	22.5 (1123)	11.1 (555)	11.7 (568)
For other purposes, but partially used to pay for healthcare cost	5.6 (280)	4.3 (216)	1.3 (64)
Type of healthcare services that the loan was used for			
Outpatient care in the past month	50.6 (710)	46.7 (360)	55.5 (350)
Inpatient care in the past 12 months	45.8 (642)	49.2 (379)	41.7 (263)
Preventive care in the past 12 months	3.6 (50)	4.2 (32)	2.9 (18)
Amount of loan (in US$) borrowed by households			
Median (IQR)	125 (50-300)	200 (100-500)	75 (30-200)
<100	37.6 (526)	24.6 (189)	53.4 (337)
101–200	21.1 (296)	21.1 (162)	21.2 (134)
201–400	18.5 (259)	22.5 (173)	13.6 (86)
≥400	22.8 (319)	31.9 (245)	11.7 (74)
Period to pay off the total (of household borrowing with interest)			
Median (in months)	__	8	__
<6 months	__	41.7 (249)	__
6–12 months	__	11.7 (70)	__
≥12 months	__	46.6 (278)	__
Current status of loan (of household borrowing)			
Still owed	78.0 (1094)	84.6 (652)	69.9 (442)
Already paid off	21.3 (299)	14.4 (111)	29.8 (188)
Missing	0.7 (10)	1.0 (8)	0.3 (2)

### Determinants of distress financing

Table 3 presents the prevalence of distress financing by household subgroup and results of the logistic regression of factors associated with such borrowing. For comparative purposes, the unadjusted odds ratios (OR) with 95% CI are also presented (the first two columns of Table 3). The multivariate logistic regression model (the last two columns of Table 3) shows that distress financing correlated with SES: households in first, second, third and fourth quintiles were found to be 6.1, 4.4, 3.4 and 3 times more likely to borrow money with interest than those of the fifth quintile (the richest group). In addition, large households (with five members or more) were 1.4 times more likely to borrow with interest compared with those having <5 members. Households with three or more members seeking outpatient care were 1.5 times more likely to borrow compared with those not seeking outpatient care. Households with two or more members seeking inpatient care at least once in the past 12 months were, respectively, 11.6 and 16 times more likely to borrow compared with those having no member doing so. However, seeking preventive care was not significantly associated with borrowing. In terms of type of provider, households seeking outpatient care from private providers only and from mixed (both private and public) providers were, respectively, 2.2 and 3.5 times more likely to borrow with interest than those seeking outpatient care from public providers only. Surprisingly, households with elderly members were less likely to borrow for healthcare compared with those with no elderly member.

**Table 3:** Factors associated with distress financing in Cambodia (2016)

	Distress financing (*N* = 4996^a^)			
	OR (95% CI)	*P*-value	AOR (95% CI)	*P*-value
Household location				
Urban	Ref.	<0.001	Ref.	
Rural	1.8 (1.4, 2.2)		0.8 (0.6, 1.0)	0.099
SES (classified into five quintiles)				
1st (poorest)	4.5 (3.3, 6.1)	<0.001	6.1 (4.2, 8.9)	<0.001
2nd	3.7 (2.7, 5.1)		4.4 (3.0, 6.3)	<0.001
3rd	3.1 (2.2, 4.2)		3.4 (2.3, 4.9)	<0.001
4th	2.9 (2.1, 3.9)		3 (2.1, 4.4)	<0.001
5th (richest)	Ref.		Ref.	
Household size				
Less than 5	Ref.	<0.001	Ref.	
5 or more	1.6 (1.4, 1.9)		1.4 (1.2, 1.7)	<0.001
Number of household members aged under 5 years				
0	Ref.	<0.001	Ref.	
1	1.6 (1.3, 1.9)		1.1 (0.9, 1.4)	0.229
2 or more	1.7 (1.4, 2.0)		1.2 (0.9, 1.5)	0.237
Number of household members aged 65 years or older				
0	Ref.	<0.001	Ref.	
1	0.7 (0.6, 0.9)		0.7 (0.6, 0.9)	0.002
2 or more	0.6 (0.4, 0.8)		0.5 (0.4, 0.8)	0.001
Number of household members sick in the past month^b^				
0	Ref.	<0.001		
1	1.8 (1.3, 2.4)		__	__
2	2.8 (2.1, 3.9)		__	__
3 or more	4.4 (3.2, 6.0)		__	__
Number of household members seeking outpatient consultation in the past month				
0	Ref.	<0.001	Ref.	
1	1 (0.8, 1.3)		1 (0.8, 1.4)	0.842
2	1.3 (1.0, 1.6)		1.2 (0.9, 1.5)	0.172
3 or more	2.1 (1.7, 2.5)		1.5 (1.2, 1.9)	0.001
Number of household members seeking inpatient care in the past 12 months				
0	Ref.	<0.001	Ref.	
1	4 (3.4, 4.7)		6.2 (0.7, 55.0)	0.102
2	7.1 (4.9, 10.3)		11.6 (1.3, 105.1)	0.029
3 or more	9.6 (5.3, 17.6)		16 (1.6, 155.9)	0.017
Number of household members seeking preventive care in the past 12 months				
0	Ref.	<0.001	Ref.	
1	1.4 (1.2, 1.7)		0.6 (0.1, 4.1)	0.632
2	2 (1.6, 2.6)		0.8 (0.1, 5.2)	0.822
3 or more	2.3 (1.5, 3.7)		0.9 (0.1, 6.2)	0.917
Type of health facility for outpatient consultation by household member(s) in the past month				
Did not use	Ref.	<0.001	Ref.	
Public	1.6 (1.0, 2.4)		1.2 (0.8, 1.9)	0.408
Private	2.2 (1.6, 3.0)		2.2 (1.6, 3.1)	<0.001
Both	4.9 (3.5, 6.7)		3.5 (2.4, 5.0)	<0.001
Type of health facility for last inpatient care by household member(s) in the past 12 months				
Did not use	Ref.	<0.001	Ref.	
Public	4.5 (3.6, 5.5)		0.6 (0.1, 5.2)	0.631
Private	4.4 (3.6, 5.3)		0.6 (0.1, 5.3)	0.644
Type of health facility for last preventive care by household member(s) in the past 12 months				
Did not use	Ref.	<0.001	Ref.	
Public	1.6 (1.4, 1.9)		1.7 (0.3, 10.6)	0.592
Private	1.7 (1.3, 2.1)		1.8 (0.3, 11.7)	0.527
OOP payment (in US$) for healthcare in the past 12 months^c^				
Median (IQR)				
<100	Ref.	<0.001	__	__
<200	2.1 (1.6, 2.9)		__	__
<300	4.3 (3.1, 6.0)		__	__
<400	3.6 (2.7, 4.8)		__	__
400	6.8 (5.4, 8.6)		__	__

a Four records were missing.

b Not included in multivariate model because highly correlated with other variables—members sought outpatient services, members sought inpatient services, and members sought preventive maternal and child care.

c Not included because this variable is just a pathway.

*N*, total number of households; OR, odd ratio; AOR, adjusted odd ratio; CI, confidence interval.

Three explanatory variables were excluded from the final model (Table 3). While OOP expenditure on healthcare is an important determinant of borrowing, it was excluded on the basis that it is an intermediary factor leading to other factors such as type of care and type of provider. Holding an IDPoor card that provides entitlement to health HEFs was found to be strongly correlated with household SES, and therefore also excluded. Finally, number of household members falling ill in the past month was also found to be highly correlated with other variables (including members seeking outpatient consultation, members seeking inpatient care and members seeking preventive care) and removed from the model. All other variables were included in the final multivariate model.

### Borrowing by HEF members

A separate analysis comparing HEF-entitled households (those holding an IDPoor card) and households with no such entitlement showed that a significantly larger proportion of the HEF-entitled group, 24.7% (290/1172) resorted to borrowing with interest to pay for healthcare than non-entitled households, 12.5% (479/3824) (chi-square test: *P* < 0.001).

## Discussion

The results show that around a third of Cambodian households in this study had healthcare-related debts. More than half of them, experienced distress financing. The average debt due to healthcare borrowing was US$125. For those who paid interest on their loan, the average debt rose to US$200, which represented ∼7% of the average rural Cambodian household’s annual income in 2012 (Tong *et al.*, 2013). Approximately three-quarters of borrowers were still paying their debt at the time of interview but this was much more common amongst those with distress financing, 85%, than households that had an interest-free loan, 70%. The reported median amount spent on healthcare was US$208 and the resulting high prevalence of borrowing in general and distress financing in particular suggests that cash flow in Cambodian society is limited. This limited cashflow may also explain the fact that interest-free loans were US$75 on average (vs US$200 for those with interest) as they primarily would have been obtained from relatives.

It is not entirely surprising that poorer households were more likely to experience distress financing than wealthier households. This is consistent with studies from Indonesia (Sparrow *et al.*, 2014), Vietnam (Nguyen *et al.*, 2012), Ethiopia (Husøy *et al.*, 2018), India (Mohanty and Kastor, 2017) and most recently in Nepal, Myanmar and India (Mohanty *et al.*, 2017). As described by Ir *et al.* (2012), poor people are most likely to be subjected to interest payments when borrowing money, often facing exorbitant rates, as the default risks are perceived as high due to limited collateral.

Our study also revealed that larger households are more likely to experience distress financing, a finding that has been reinforced elsewhere, including Kenya, Tanzania, Togo, Iran and Brazil (Brinda, 2014; Fazaeli *et al.*, 2015; Luiza *et al.*, 2016; Barasa *et al.*, 2017; Atake and Amendah, 2018) that also found households size to be a major determinant for catastrophic health expenditure. This has been explained by higher healthcare seeking frequency, increased dependency ratios and insufficient financial risk protection by health insurance schemes among larger families (ibid). In contrast, a study from Kenya found that larger households located in a slum area were less likely to experience catastrophic health expenditure (Buigut *et al.*, 2015) due to the fact that they included more working members. In Turkey (Yardim *et al.*, 2010), household size was shown to have no effect on catastrophic health expenditure. For this study, the most likely explanation for significant distress financing amongst larger households is the higher number of members in these households which can increase the need for healthcare. This explanation is plausible given that the frequency of care seeking (both for hospitalizations and ambulatory care) was also shown to be a major determinant of distress financing in this study and elsewhere (Binnendijk *et al.*, 2012; Quintussi *et al.*, 2015; Prinja *et al.*, 2016).

Households seeking care from private providers were also found to be more susceptible to distress borrowing compared with those that sought care only in the public sector. Ambulatory care was mainly sought in the private sector (64%), while nearly equal proportions were admitted at public (10.5%) and private health facilities (12%). In many LMICs, the private health sector is poorly regulated and people seeking care in this sector run the risk of dealing with poorly qualified providers who often provide substandard care and subject patients to needless and expensive treatments (Morgan *et al.*, 2016). Such phenomenon can result in patients being pushed into a ‘medical poverty trap’ whereby the uncontrolled growth of the private sector coupled with increased OOP expenses for healthcare reduce access to effective quality treatment and exacerbate long-term poverty (Whitehead *et al.*, 2001). The scale of OOP costs in the private sector was highlighted in a recent study from India where admission to a public hospital without insurance coverage was shown to be cheaper than admission to a private hospital with insurance coverage (Ranjan *et al.*, 2018). Despite the risks and high costs associated with the private health sector, the majority of Cambodians continue to initiates care seeking with such providers (Dalal *et al.*, 2017). The eventual disastrous impact of such care seeking behaviour has been documented by Van Damme *et al.* (2004) who found that those seeking care from private providers for children with dengue fever paid about 13 times more than in the public sector and just over one-third had fully repaid their loan a year later.

Surprisingly, we did not observe an effect of older household members on distress financing. This is in contrast to an earlier study from Cambodia that showed such households were at considerable risk for catastrophic health expenditure (Jacobs *et al.*, 2016). Similar findings have been reported in Vietnam (Van Minh *et al.*, 2013; Kien *et al.*, 2016), India (Shahrawat and Rao, 2012; Pandey *et al.*, 2018) and China (You and Kobayashi, 2011; Kumar *et al.*, 2015). Explanations put forward for this higher incidence of catastrophic health expenditure include the increased dependency of old people together with their greater likelihood of suffering diseases and disabilities (Brinda *et al.*, 2012; Ha *et al.*, 2015). Parents cohabiting with their children is common in Southeast Asia (Teerawichitchainan *et al.*, 2015) and in the absence of mature social protection programmes older people can impose a considerable financial burden (Sousa *et al.*, 2009). For example, in Vietnam, the extra costs to a household of a person with a disability amounted to about 9% of its annual income (Van Minh *et al.*, 2015) while the indirect cost associated with caring for such a person was about four to five times higher (Riewpaiboon *et al.*, 2014). Similarly, a recent cohort study in three middle-income countries (Guerchet *et al.*, 2018) found that, over time, the income of households with elders requiring assistance became lower than in households with older people not needing care. The curious lack of effect of elderly household members on distress financing in this study warrants further investigation.

Although HEF should reduce the amount of OOP expenditure for healthcare (Flores *et al.*, 2013), our finding that many households with HEF entitlements still experience distress financing is regrettable and confirms earlier findings (Jacobs *et al.*, 2007b, 2016). A reason for this may be related to the fact that many HEF beneficiaries still seek care in the private health sector where health services are not covered by HEFs (Jacobs *et al.*, 2018). Reasons for such behaviour include: uncertainties about HEF entitlements; cost of transport to health facilities (especially the cost of health centres which is not reimbursed if the condition does not justify referral to a hospital); having to pay for transport in advance even if it is to be covered later by the HEF; perceived uncertainty of staff presence at health centres and generally poor quality of care; restricted opening hours of health centres; waiting times at public facilities and; a preference for injections and transfusions that are not provided at public facilities over oral medication (Jacobs *et al.*, 2007a; Noy and Saign, 2011). Our research highlights important gaps in the effectiveness of the HEF, particularly the need for better targeting of households and population groups who may be vulnerable to distress financing.

Finally, preventive care was not associated with distress financing. Over the past decade, Cambodia has made remarkable advances in the delivery of maternal and child health services as well as promoting equity of uptake (Dingle *et al.*, 2013) through a variety of health financing interventions such as vouchers for reproductive health, a midwifery incentive scheme, and internal and external contracting (Ir *et al.*, 2010, 2015; Jacobs *et al.*, 2010; Richard *et al.*, 2010; Fazaeli *et al.*, 2015). Preventive care is increasingly sought in the private sector as exemplified by the fact that 32.6% of the reported consultations for preventive care occurred in private facilities. Preventive care if sought in the private sector in Cambodia is not free of charge. However, preventive services in the public sector are provided at minimal to no cost to the user. The absence of distress financing for preventive services in this study may be because the better-off population with cash at hand seek such services in private health facilities while the poor seek such services in the public sector.

Thus, it appears that the Cambodian health system has benefited from making preventive services accessible to the population. Unfortunately, the same cannot be said for curative care as exemplified by the observed prevalence of distress financing for households experiencing multiple curative encounters during the assessed period. To minimize distress financing associated with curative care, it will therefore be necessary to improve the coverage and comprehensiveness of existing social health protection schemes and to shift care seeking away from unscrupulous private health providers. Thailand took a similar approach in its pursuit of universal health coverage (UHC) by redirecting care seeking towards the public health sector once the entire population was covered by health insurance (McPake and Hanson, 2016). However, such coverage takes time to achieve which is why it may be opportune to also ensure better targeted interventions for those in higher need of effective financial risk protection, which is the case of Cambodia includes poorer and larger households. For example, Mitra *et al.* (2016) found in Vietnam that poor households were able to cope with financial health shocks without borrowing, something they ascribe to improved targeting and effectiveness of social health protection programmes in Vietnam.

### Limitations

This study has some limitations. Firstly, we only consider borrowing with interest and not the sale of assets or interest-free borrowing thereby potentially underestimating the true extent of indebtedness. However, the focus on interest-related borrowing may be justified on the grounds that it concerns a highly vulnerable group of households that are likely have little social capital, are unable to approach friends/neighbours/relatives for financial support and are thus most prone to distress financing. As mentioned, social capital is an important determinant of access to soft loans or borrowing at minimal or no interest. We did not include this concept in our framework or model due to the complex nature of this phenomenon and the data required to accurately measure it. Thus social capital is a complex construct which cannot be measured by proxies. This explains, to some extent, why the phenomenon has been assessed mainly in high-income countries and the few generic questionnaires that exist to assess it have not been validated in low- and middle-income settings (Harpman *et al.*, 2002; Story, 2013).

We may have overestimated the incidence of distress financing, as poor people in general tend to borrow more than the direct costs incurred with care seeking (Jacobs *et al.*, 2007b; Ir *et al.*, 2012). In other words, borrowing is sometimes taken as an opportunity to pay off other debts or use money for other reasons. We also did not assess the effect of non-communicable diseases on distress financing. Such conditions are common in Southeast Asia, including Cambodia (Dans *et al.*, 2011) and impose considerable financial hardship, especially amongst poorer households (Jan *et al.*, 2018). As loans were mainly used to pay for outpatient consultations occurring primarily with private health providers, the main costs may not have been associated with a single episode of care seeking but as part of a series of consultations, common for patients with chronic non-communicable diseases. Such expenses are not captured by cross-sectional surveys using a 1-month recall period as in our study but would be best determined using panel data (Kankeu *et al.*, 2013). As mentioned, we did not differentiate between care seeking for an acute illness or a chronic condition. In light of these limitations, fully capturing determinants of distress financing using cross-sectional surveys remains challenging. One way of addressing this issue is to expand the recall period for outpatient consultations related to non-communicable diseases to 1 year (although this may increase recall bias) and by incorporating valid proxy measures for social capital.

## Conclusion

This study provides useful evidence for health financing policy and social health protection in Cambodia where financial risk protection is limited and distress financing—measured as borrowing with interest—is common. The financial burden on households appears to be considerable, evidenced by the fact that over three-quarters of the households with distress financing in this study remained in debt 1 year after taking out the loan, beyond the average intended lending period of 8 months. Poor households were shown to be at particular risk of distress financing which can push them into heavy indebtedness and deeper poverty. This result sends a clear message that the level of financial protection currently offered to poor households under the HEF and through other financing reforms targeting the poor, is not sufficient to achieve financial risk protection necessary to move Cambodia towards UHC. In order to ensure effective financial risk protection, Cambodia should establish a more comprehensive and effective social health protection scheme that provides maximum population coverage and prioritizes services for populations at risk of distress financing, especially poorer and larger households.
